# Radiomics Signature as a Predictive Factor for EGFR Mutations in Advanced Lung Adenocarcinoma

**DOI:** 10.3389/fonc.2020.00028

**Published:** 2020-01-31

**Authors:** Duo Hong, Ke Xu, Lina Zhang, Xiaoting Wan, Yan Guo

**Affiliations:** ^1^Department of Radiology, The First Hospital of China Medical University, Shenyang, China; ^2^GE Healthcare, Shanghai, China

**Keywords:** advanced lung adenocarcinoma, tomography, epidermal growth factor receptor, mutation, radiomics

## Abstract

**Purpose:** To develop and validate a radiomic signature to identify EGFR mutations in patients with advanced lung adenocarcinoma.

**Methods:** This study involved 201 patients with advanced lung adenocarcinoma (140 in the training cohort and 61 in the validation cohort). A total of 396 features were extracted from manual segmentation based on enhanced and non-enhance CT imaging after image preprocessing. The Lasso algorithm was used for feature selection, 6 machine learning methods were used to construct radiomics models. Receiver operating characteristic (ROC) curve analysis was applied to evaluate the performance of the radiomic signature between different data and methods. A nomogram was developed using clinical factors and the radiomics signature, then it was analyzed based on its discriminatory ability and calibration. Decision curve analysis (DCA) was implemented to evaluate the clinical utility.

**Results:** Ten features for contrast data and eleven features for non-contrast data were selected through LASSO algorithm. The performance of the radiomics signature for contrast images was better than that for non-contrast images in all of the 6 different machine learning methods. Finally, the best radiomics signature was built with logistic regression method based on enhanced CT imaging with an area under the curve (AUC) of 0.851 (95% CI, 0.750 to 0.951) in the validation cohort. A nomogram was developed using the radiomics signature and sex with a C-index of 0.908 (95%CI, 0.862 to 0.954) in the training cohort and 0.835 (95% CI, 0.825 to 0.845) in the validation cohort. It showed good discrimination and calibration (Hosmer-Lemeshow test, *P* = 0.621 for the training cohort and *P* = 0.605 for the validation cohort).

**Conclusion:** Radiomics signature can help to distinguish between EGFR positive and wild type advanced lung adenocarcinomas.

## Introduction

Lung cancer is one of the most common malignant tumors in the world and the leading cause of cancer-related death worldwide (1). The World Health Organization (WHO) divides lung cancer into two major categories: non-small cell lung cancer (NSCLC), representing more than 85% of all cases, and small cell lung cancer (SCLC). Adenocarcinoma in NSCLC is the major histological subtype, accounting for almost half of all lung cancer cases (2). The 5-year survival rate is >50% when the disease is still localized; however, 75% of cases are diagnosed at an advanced stage with unresectable lesions (3).

Over the last decade, advances in molecularly targeted drugs for thoracic oncology have led to a new emphasis on accurate analyses of biomolecular markers in a subset of lung adenocarcinoma (4). Patients with advanced lung adenocarcinoma harboring epidermal growth factor receptor (EGFR)-activating mutations showed a significant progression-free survival (PFS) benefit with reduced side effects by treatment with tyrosine kinase inhibitor (TKIs) (5). TKI therapy had already been used as first-line systemic therapy before chemotherapy (6, 7). Biopsy is the only widely used means to identify mutations of EGFR in unresectable lesions, but some patients refuse the procedure due to the risk of hemorrhage and pneumothorax. Furthermore, it is difficult to obtain tissue samples from inaccessible locations in some cases. Therefore, automatic, non-invasive, and cost-effective alternatives are desired (8). Radiomics refers to the systematic extraction and analysis of features from digital medical images with the intent of creating mineable databases to aid in diagnosis and treatment. Radiogenomics even involves specific features connecting genomic phenotypes and radiological images. The aim of this study was to develop a radiogenomic approach to identify EGFR mutations in advanced lung adenocarcinoma non-invasively.

## Materials and Methods

### Patients

**Figure 1:**
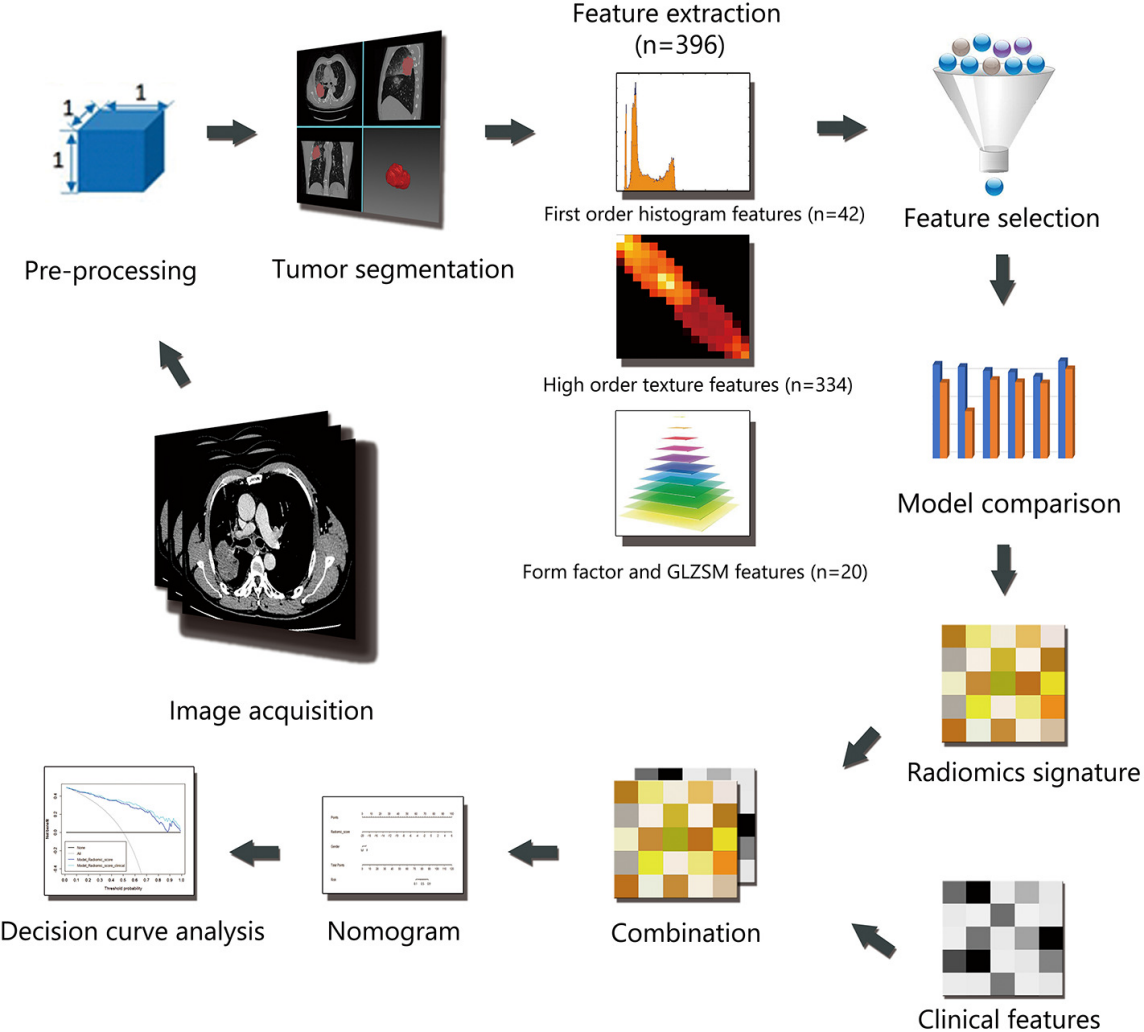
Workflow of the radiomic analysis.

Institutional review board approval was obtained for this retrospective study, and with a waiver for the informed consent requirement. Consecutive patients (*n* = 449) with advanced lung adenocarcinoma who were admitted to the hospital from January 2014 to January 2016 were enrolled in this retrospective study. All cases were histologically confirmed by transthoracic biopsy and classified as stage IIIB-IV according to the Eighth Edition of the Lung Cancer Stage Classification (9). EGFR mutations in exons 18, 19, 20, and 21 were detected using human EGFR gene mutations detection kit (AmoyDx, China) via Amplification Refractory Mutation System (ARMS) real-time Polymerase Chain Reaction (PCR) technology. A total of 248 patients were excluded based on the following exclusion criteria: [1] examined by an unassigned CT scanner (*n* = 105); [2] received previous anticancer therapy or with other types of cancer (*n* = 19); [3] no EGFR mutation analysis available (*n* = 81); [4] difficulty in drawing regions of interest (ROIs) (*n* = 43). Finally, 201 patients were included in the study. The clinical data collected for analysis included sex, age, smoking status, and stage. The patients were randomly divided into two individual cohorts for training and validation at a ratio of 7:3. The workflow of the radiomic analysis is illustrated in Figure 1.

### Image Acquisition

Contrast-enhanced computed tomography (CT) images were acquired at our hospital using either a Toshiba Aquilion One (Toshiba Medical Systems) or Phillips Brilliance iCT (Philips Medical Systems) system. The scanning parameters were as follows: 120 kVp; 100-200 mAs; detector collimation of 64× or 128× 0.625 mm; field of view of 350 × 350 mm; and matrix of 512 × 512. After routine CT, a dose of 85 mL non-ionic iodinated contrast material (350 mg iodine/mL, Omnipaque, GE Healthcare) was injected into the antecubital vein at a rate of 3.0 mL/s using an automated injector (Ulrich CT Plus 150, Ulrich Medical). CT scanning was performed again with a 25-second delay after the injection. All images were reconstructed at a slice thickness of 2 mm. Contrast and non-contrast images were retrieved separately from the Picture Archiving and Communication System (PACS) workstation (IMPAX, AGFA).

### Image Preprocessing

**Figure 2:**
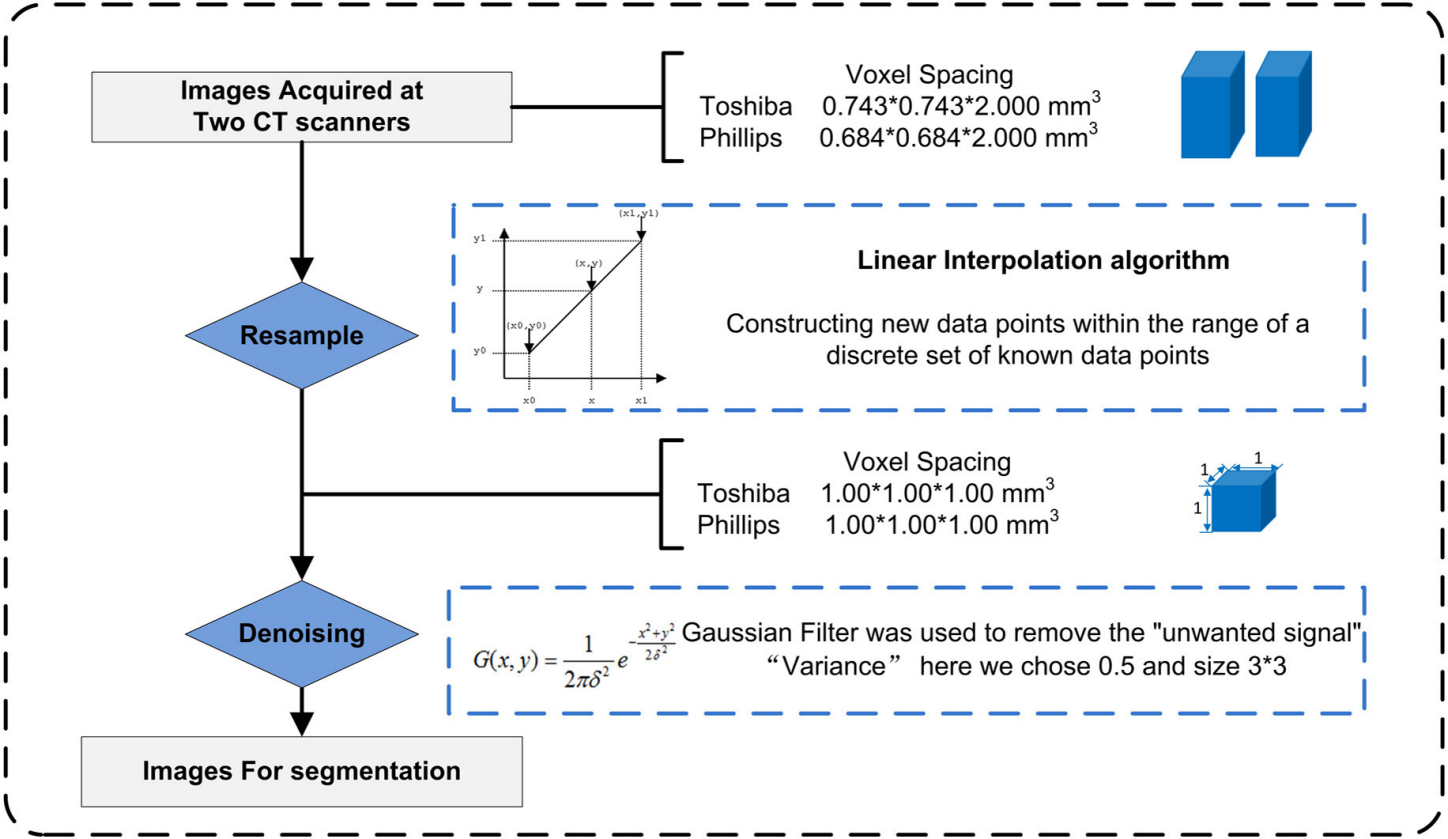
Image preprocessing.

Due to the use of different CT scans, image preprocessing (Figure 2) before segmentation and feature extraction was performed to improve the robustness of the radiomic features. The process included two steps: Step 1. To eliminate the intrinsic dependency on voxel size for the radiomic features, a resampling method with a linear interpolation algorithm was used to normalize the voxel size. Meanwhile, higher-order texture analysis features, such as GLCM and GLRLM features, were derived from different directions (also called “angles”) and different scales (denoted here as “offsets”); thus, the anisotropic voxels scanned at 0.743 mm*0.743 mm*2.000 mm or other size were resampled to form isotropic voxels, i.e., 1.000 mm*1.000 mm*1.000 mm. Step 2. A Gaussian filter was used to remove “unwanted signals”, i.e., noise beyond the scope of the (μ ± 3σ) CT values. The gray level was consistent across the different scanners, so gray level normalization was not used here.

### Tumor Segmentation

ROIs were manually contoured along the boundaries of the tumor layer by layer in reference to images in both the mediastinum and lung windows. Segmentation was strictly performed by a chest radiologist (W.XT.) with 7-year experience in lung CT using ITK-Snap (version 3.4.0, www.itk-snap.org) software and confirmed by another chest radiologist (H.D.) with 13-year experience. Both radiologists were blinded to the diagnosis and EGFR mutation status.

### Feature Extraction

Four types of radiomic features were extracted from both contrast and non-contrast CT images, and the details are shown in Figure S1. Features based on the three-dimensional volume of interest (3D VOI) were generated automatically using in-house software (Artificial Intelligence Kit, A.K., GE Healthcare).

### Feature Selection

Some features might contribute to the positive performance of classification while others might add noise to it (10). The least absolute shrinkage and selection operator (LASSO) algorithm, which is suitable for high-dimensional low-sample size data with the problem of collinearity (11, 12), was used to select effective and predictable features in the training cohort after data split. Features with nonzero coefficients were chosen based on 10-fold cross-validation.

### Model Construction

After feature selection, 6 machine learning methods were used to construct models which include NBC (Naive Bayesian Classifier), KNN (K-Nearest Neighbor), RF (Radom Forest), SVM (Support Vector Machine), DT (Decision Tree), LR (Logistic Regression). Their predictive performance was measured by using area under the curve (AUC) of receiver operating characteristic (ROC) curve analysis in the validation cohort. First, AUC of each model in contrast and non-contrast data were compared, and inferior data was abandoned, then in superior data, the optimal model was chosen for further analysis.

### Nomogram Construction

The nomogram was constructed based on multivariable logistic regression analysis. Clinical factors and radiomics signature were included in a nomogram model for predicting EGFR mutations in the training cohort. The discriminative power of the model was evaluated by Harrell's concordance index (C-index) with 95% confidence intervals in both cohorts. The calibration curve was plotted to explore the predictive accuracy of the model. Decision curve analysis (DCA) was implemented to evaluate the clinical usefulness by quantifying the net benefits of the nomogram model in both the training and validation cohorts.

### Statistical Analysis

All statistical tests were performed using R statistical software version 3.5.2. The “glmnet” package was used for executing the LASSO algorithm. For the baseline characteristic analyses, quantitative data were compared using Student's *t*-test, and categorical data were compared using the χ2 test. All statistical tests were two-tailed, and *p* < 0.05 indicated a significant difference.

## Results

The baseline clinical characteristics of the training and validation cohorts are listed in Table 1. There was no significant difference between training and validation cohorts in overall distribution of age, sex, smoking status or stage.

**Table 1:** Demographic data of patients in the training and validation cohorts.

**Variable**	**Training cohort**			**Validation cohort**			***p***
	**Mutant**	**Wild type**	***p***	**Mutant**	**Wild type**	***p***	
Age (y, mean ± SD)	58.24 ± 11.05	57.93 ± 8.43	0.85	59.23 ± 7.62	57.07 ± 8.38	0.276	0.929
Sex, *n* (%)			0.007^*^			0.149	0.437
Male	28(40.0)	44(62.9)		15(46.6)	20(66.7)		
Female	42(60.0)	26(37.1)		16(53.4)	10(33.3)		
Smoking Status, *n* (%)			0.003^*^			0.09	0.396
Smoker	13(18.6)	29(41.4)		8(25.8)	14(46.7)		
Never smoker	57(81.4)	41(58.6)		23(74.2)	16(53.3)		
Stage, *n* (%)			0.002^*^			0.119	0.103
III	4 (5.7)	17 (24.3)		5 (16.1)	10 (33.3)		
IV	66 (94.3)	53 (75.7)		26 (83.9)	20 (66.7)		
Radiomic score, median (interquartile range)	1.42 (0.57 to 2.46)	−1.63 (−2.88 to 0.42)	<0.001^*^	1.01 (−0.53 to 2.21)	−1.93 (−4.47 to −1.22)	<0.001^*^	0.145

* *P-value < 0.05*.

A total of 396 features were extracted. In the training cohort, 10 features for contrast images and 11 features for non-contrast images were evaluated to construct models through LASSO algorithm (Figure S2, Table S1).

**Figure 3:**
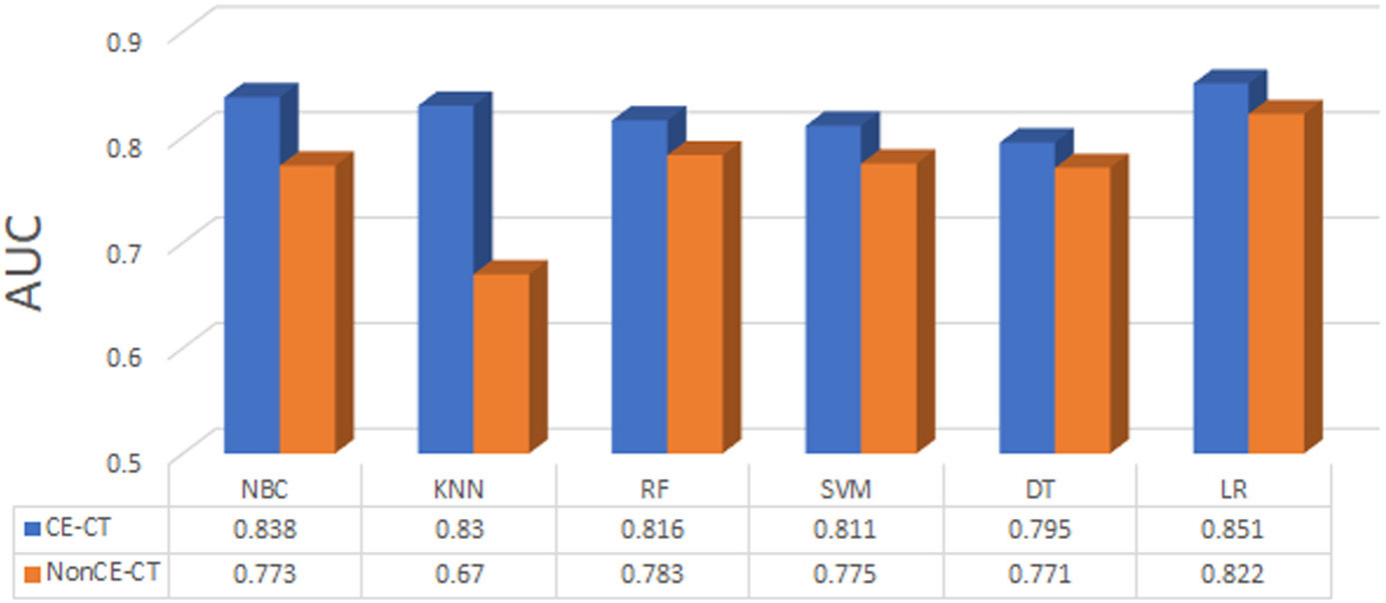
The predictive performance of all machine learning methods based on contrast (CE-CT) and non-contrast (nonCE-CT) data.

The predictive performance of all six models based on contrast and non-contrast data were described in Figure 3. The predictive performance of all six models based on contrast and non-contrast data were described in Figure 3. Although there was no significant difference by Delong test in all results, the value of AUC in contrast images was better than non-contrast images in all models, hence, the non-contrast data was excluded from further analysis. The machine learning method of LR which could assign each patient a radiomic score (rad-score) obtained a better value than other models, therefore, the nomogram was built based on the LR model in contrast data.

**Figure 4:**
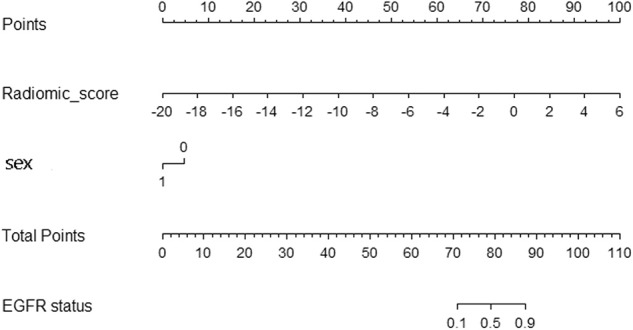
Radiomic nomogram. In the training cohort, the nomogram incorporated the radiomic signature and sex.

Table 2 shows the results of multivariable logistic regression analysis including sex, age, smoking status, and rad-score. Sex and rad-score appeared to be independent prognostic predictors of mutations in this model. The model that incorporated the above independent predictors is presented as the nomogram (Figure 4). The model showed a favorable C-index of 0.908 (95% CI, 0.862 to 0.954) in the training cohort and 0.835 (95% CI, 0.825 to 0.845) in the validation cohort.

**Table 2:** Multivariable logistic regression for nomogram construction.

	**Coefficient**	**Odds ratio**	**95% CI**		***p***
			**Lower**	**Upper**	
Intercept	−0.734				0.049^*^
Radiomic score	−1.023	0.359	0.256	0.504	<0.001^*^
Sex^#^	1.139	3.124	1.116	9.742	0.030^*^
Smoking status^†^	0.450	1.569	0.521	4.726	0.424

# *Male was denoted as 0, and Female as 1. The Odds Ratio was 3.124 means that female showed higher likelihood of EGFR (+)*.

† *Smoker was denoted as 0, and Never smoker as 1. The Odds Ratio was 1.569 means that Never smoker showed higher likelihood of EGFR (+)*.

* *P-value < 0.05, which showed significance*.

**Figure 5:**
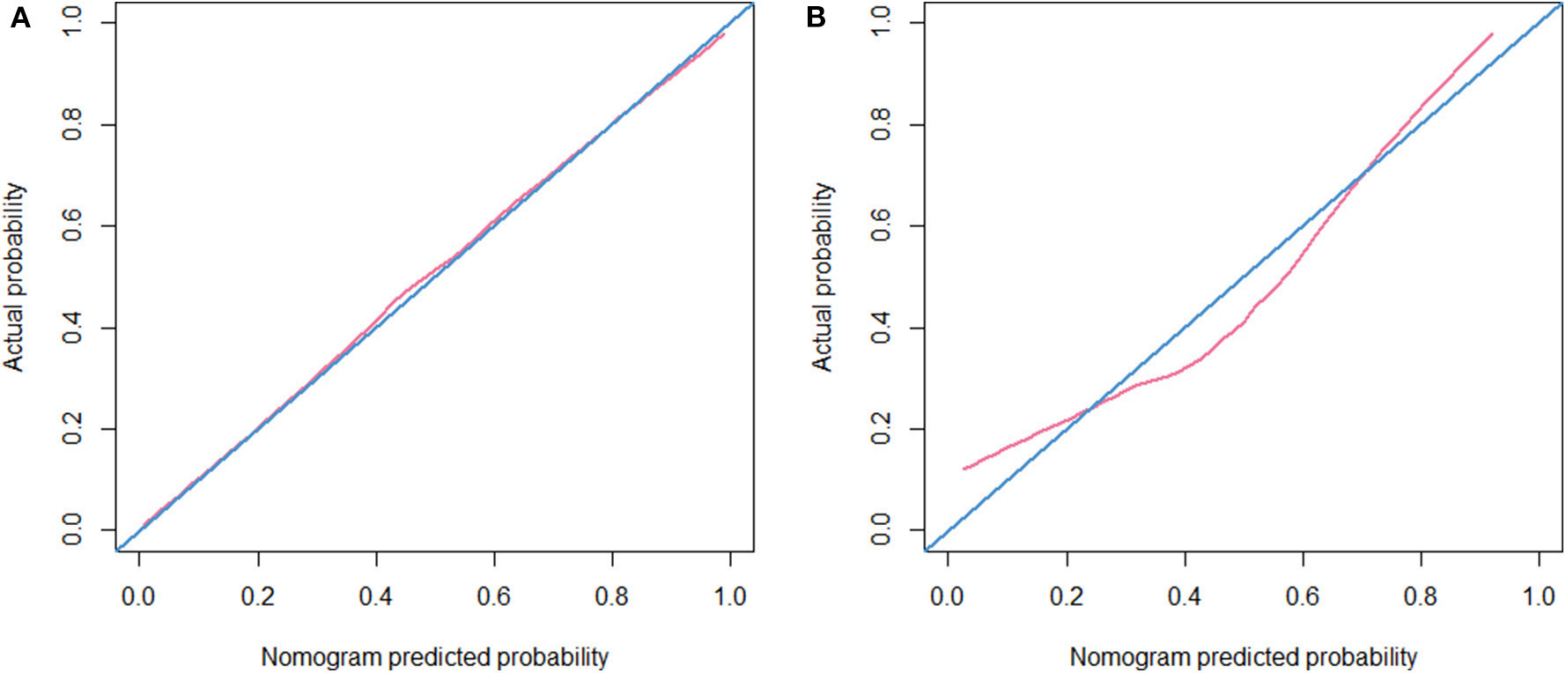
Calibration curve. **(A)** Calibration curve of the nomogram in the training cohort. **(B)** Calibration curve of the nomogram in the validation cohort.

The calibration curve of the radiomic nomogram for the probability of EGFR mutations demonstrated good agreement between the predicted and observed results in both cohorts (Figure 5). The Hosmer-Lemeshow test showed no significant statistical difference between calibration curves and ideal curves (*P* = 0.621 for the training cohort and *P* = 0.605 for the validation cohort).

**Figure 6:**
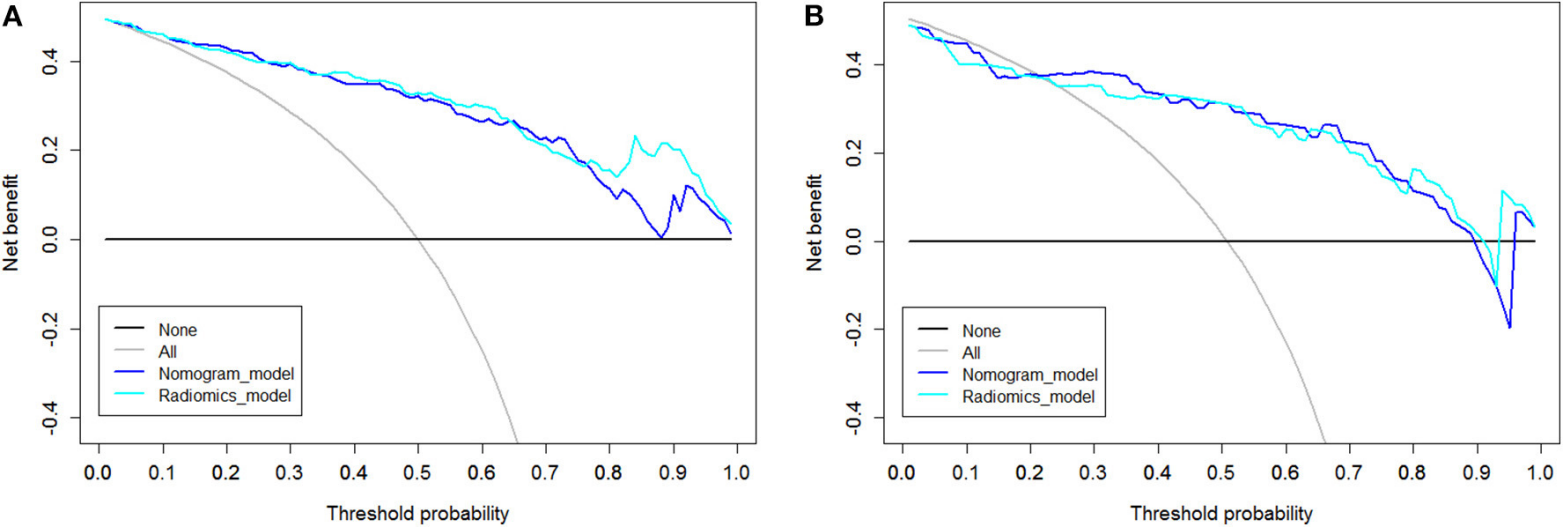
Decision curve analysis (DCA). The y axis represents the net benefit, which was determined by calculating the difference between the expected benefit and the expected harm associated with each proposed model [net benefit = true-positive rate (TPR) – (false-positive rate (FPR)× weighting factor), where the weighting factor = threshold probability/ (1-threshold probability)]. The gray line represents the assumption that all tumors were EGFR (+) (the treat-all scheme). The black line represents the assumption that all tumors were EGFR (-) (the treat-none scheme). **(A)** DCA in the training cohort. Using the radiomic model and the nomogram model to predict the EGFR status added more benefit than using the treat-all scheme or the treat-none scheme at any given threshold probability. **(B)** DCA in the validation cohort. For threshold probabilities >20%, using the radiomic model and the nomogram model to predict the EGFR status added more benefit than using the treat-all scheme or the treat-none scheme.

DCA was performed for the radiomic model (light blue line) and nomogram model (dark blue line) as shown in Figure 6. Using the radiomic model and the nomogram model to predict the EGFR status added more benefit than using the treat-all scheme or the treat-none scheme at any given threshold probability in the training cohort. For threshold probabilities > 20%, using the radiomic model and the nomogram model to predict the EGFR status added more benefit than using the treat-all scheme or the treat-none scheme in the validation cohort.

## Discussion

The NCCN (2019, v3) recommended that testing for EGFR mutations should be applied in patients with non-squamous NSCLC or NSCLC NOS (not otherwise specified) so that patients with this genetic abnormality can receive effective treatment with targeted agents. Although patients with advanced adenocarcinoma benefit most from TKIs, accessibility to obtain transbronchial or transthoracic biopsy samples is not always satisfactory or safe in these patients. The adverse event rate in thoracic biopsy was reported to be 17.1% (13), and sufficient tissue for molecular analysis can only be obtained in 20–50% of NSCLC patients, even in large well-designed clinical trials (14). In addition, the heterogeneity of the tumor may mislead the clinical decision (15, 16).

We developed and validated a radiomics signature-based nomogram for the non-invasive detection of EGFR mutations in patients with advanced adenocarcinoma through preprocessing, parameters screening and model building from CT images. In the validation cohort, the AUC of radiomics signature was 0.851 (95% CI, 0.750 to 0.951). Previous studies have demonstrated such correlations in all stages of peripheral lung adenocarcinoma (17, 18), with AUC of 0.709 (95% CI, 0.645 to 0.766) and 0.751 (95% CI, 0.631 to 0.848), respectively. For early-stage resectable adenocarcinoma, the detection is less important, whereas for advanced-stage patients with EGFR mutations, TKIs are the first-line standard modality for the treatment today (19), so the detection is urgently needed. Thus, it is of greater significance to establish relatively inexpensive and safe imaging biomarker for the advanced-stage patients to help making treatment decision. However, stage selection brought limitation at the same time. The signature could not act as an alone biomarker in patients with unknown pulmonary nodules and it is also a time-consuming thing to stage before using the biomarker.

Previous articles on pulmonary tumor radiomics were generally based on non-contrast CT images (20–22). Some studies have used contrast images alone (23, 24), and some have used both, but no comparisons or descriptions regarding which type of image is better for further analysis have been reported (25). In this study, we managed contrast and non-contrast data separately and compared their diagnostic value using ROC curve analysis; we finally chose the contrast data for subsequent analysis. This result is consistent with clinical applications. Contrast-enhanced CT can be used to better delineate and define tumor regions in relationship to surrounding structures than non-contrast CT, and also demonstrates the increased vascularity that occurs within malignancies and provides additional information on the tumor's physiology and active blood supply. All of this information is reflected by radiomic features, leading to better models.

However, some limitations to this work still exist. First, although image acquisition was confined to two CT systems and all the images were preprocessed before segmentation, differences between devices may influence the results. Second, in the baseline clinical characteristics, there was no significant difference in the overall distribution of age, sex, smoking status or stage between the training and validation cohorts, thus we believed that there was no bias for the training and validation cohorts. But when taking into consideration the distribution in mutant and wild-type EGFR patients, sex and smoking status showed significant differences between the two groups in the training cohort but no significance in the validation cohort, which we considered may due to the small sample size in the validation cohort. Third, less sample size and lack of external validation of the model, more multicenter studies and prospective studies should be carried out to increase the generalizability and robustness of the radiomic findings. Fourth, all samples were obtained through biopsy. They were smaller than those obtained by surgery, which could better represent the tumor heterogeneity. Further studies may also include testing for cell-free tumor DNA (ctDNA) and circulating tumor cells (CTCs) to ensure the homogeneity of mutations (14).

## Conclusion

In conclusion, radiomics signature can help to distinguish between EGFR positive and wild type advanced lung adenocarcinomas. Compared with non-contrast CT, contrast-enhanced CT provided more value for radiomic predication.

## Data Availability Statement

The raw data supporting the conclusions of this article will be made available by the authors, without undue reservation, to any qualified researcher.

## Ethics Statement

The studies involving human participants were reviewed and approved by the Institutional Review Board of the First Hospital of China Medical University. Written informed consent for participation was not required for this study in accordance with the national legislation and the institutional requirements.

## Author Contributions

DH conceived of the project, performed the experiments and wrote the paper. YG and XW analyzed the data. KX and LZ provided expert guidance and reviewed the manuscript. All the authors gave the final approval of the manuscript.

### Conflict of Interest

YG was employed by the company GE Healthcare, China. The remaining authors declare that the research was conducted in the absence of any commercial or financial relationships that could be construed as a potential conflict of interest.

## Supplementary Material

Figure S1 Details of four types of radiomic features extracted from CT images.

Figure S2 Feature selection using the LASSO algorithm in the training cohort for contrast images. **(A)** The 10-fold cross-validation process was repeated to select the optimal penalization coefficient lambda. The value of lambda yielded the minimum average binomial deviance that was used to select features. **(B)** LASSO coefficient profile plot of the 396 features against log(lambda); the optimal lambda resulted in 10 non-zero features.

Table S1 The formulas for calculation of the radiomics signature.
