# Supplementary figures and images for: Radiomics Signature as a Predictive Factor for EGFR Mutations in Advanced Lung Adenocarcinoma
Source: Front Oncol. 2020 Jan 31;10:28. doi: 10.3389/fonc.2020.00028 (PMC7005234; doi:10.3389/fonc.2020.00028)

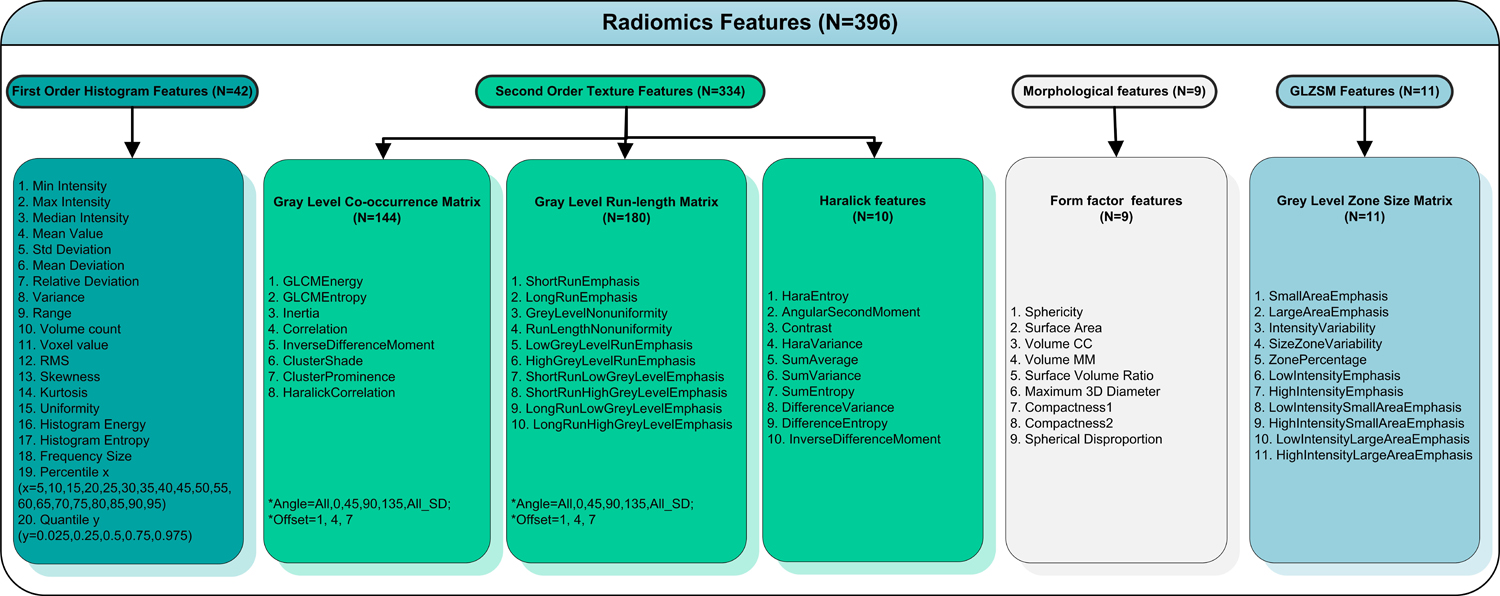

Supplement: Figure S1 — Details of four types of radiomic features extracted from CT images. [file Image_1.JPEG]

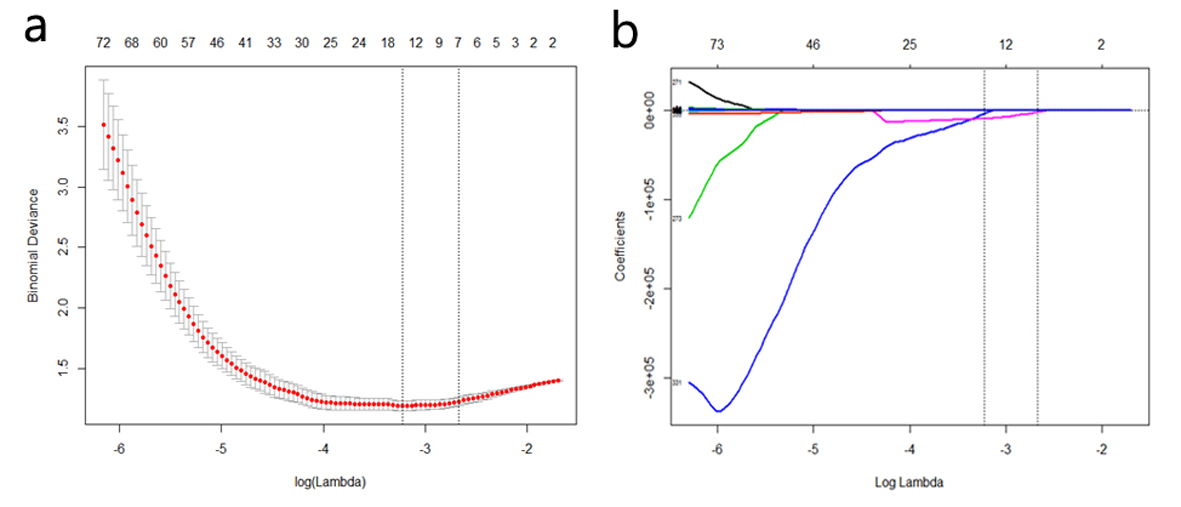

Supplement: Figure S2 — Feature selection using the LASSO algorithm in the training cohort for contrast images. (A) The 10-fold cross-validation process was repeated to select the optimal penalization coefficient lambda. The value of lambda yielded the minimum average binomial deviance that was used to select features. (B) LASSO coefficient profile plot of the 396 features against log(lambda); the optimal lambda resulted in 10 non-zero features. [file Image_2.JPEG]
