# Supplementary material for: Radiomics Signature as a Predictive Factor for EGFR Mutations in Advanced Lung Adenocarcinoma
Source: Front Oncol. 2020 Jan 31;10:28. doi: 10.3389/fonc.2020.00028 (PMC7005234; doi:10.3389/fonc.2020.00028)
Supplement: Table S1 — The formulas for calculation of the radiomics signature. [file Table_1.DOCX]

Radiomics signature for Contrast images =-7.71+

4.31*10-3*Range+

7.53*10-1*Skewness-

2.82*102* InverseDifferenceMoment_AllDirection_offset4_SD+

2.21*10-8*HaralickCorrelation_AllDirection_offset4_SD+

2.61*102*InverseDifferenceMoment_AllDirection_offset7_SD+

6.07*10-1*GLCMEntropy_angle90_offset7+

1.35*10-4*ShortRunHighGreyLevelEmphasis_angle0_offset4-

2.87*105*LowGreyLevelRunEmphasis_AllDirection_offset7_SD-

3.01*104*ShortRunLowGreyLevelEmphasis_AllDirection_offset7_SD-

6.01*Sphericity

Radiomics signature for non-Contrast images =3.12-

-3.06*10-3*Min Intensity-

8.02*10-3 *Max Intensity+

3.45*10-8*HaralickCorrelation_AllDirection_offset1_SD-

3.59* GLCMEntropy_AllDirection_offset4_SD-

1.10*102*InverseDifferenceMoment_AllDirection_offset4_SD+

4.51*10-1*GLCMEntropy_AllDirection_offset7+

9.21*10-3*HighGreyLevelRunEmphasis_AllDirection_offset1_SD-

1.04*104*ShortRunLowGreyLevelEmphasis_AllDirection_offset1_SD-

2.29*104*ShortRunLowGreyLevelEmphasis_AllDirection_offset4_SD+

2.67*10-5*RunLengthNonuniformity_angle0_offset4-

11.20*Sphericity
